# Network Pharmacology Integrated with Molecular Docking Explores the Mechanisms of Naringin against Osteoporotic Fracture by Regulating Oxidative Stress

**DOI:** 10.1155/2021/6421122

**Published:** 2021-09-20

**Authors:** Xiang Yu, Peng Zhang, Kai Tang, Gengyang Shen, Honglin Chen, Zhida Zhang, Wenhua Zhao, Qi Shang, Guangye Zhu, Riwei Tan, Yanchi Gan, You Zhang, De Liang, Hui Ren, Xiaobing Jiang, Bengen Zhou

**Affiliations:** ^1^The First Affiliated Hospital of Guangzhou University of Chinese Medicine, Guangzhou 510405, China; ^2^Guangzhou University of Chinese Medicine, Guangzhou 510405, China; ^3^Lingnan Medical Research Center of Guangzhou University of Chinese Medicine, Guangzhou 510405, China

## Abstract

Naringin (NG), as the most abundant component of *Drynariae Rhizoma* (Chinese name: Gusuibu), has been proved to be an antioxidant flavonoid on promoting osteoporotic fracture (OF) healing, but relevant research is scanty on the underlying mechanisms. We adopted target prediction, protein-protein interaction (PPI) analysis, Gene Ontology (GO) analysis, Kyoto Encyclopedia of Genes and Genomes (KEGG) analysis, and molecular docking to establish a system pharmacology database of NG against OF. Totally 105 targets of naringin were obtained, including 26 common targets with OF. A total of 415 entries were obtained through GO Biological Process enrichment analysis (*P* < 0.05), and 37 entries were obtained through KEGG pathway enrichment analysis with seven signaling pathways included (*P* < 0.05), which were primarily concerned with p53, IL-17, TNF, estrogen, and PPAR signaling pathways. According to the results of molecular docking, naringin is all bound in the active pockets of the core targets with 3–9 hydrogen bonds through some connections such as hydrophobic interactions, Pi-Pi stacked interactions, and salt bridge, demonstrating that naringin binds tightly to the core targets. In general, naringin may treat OF through multiple targets and multiple pathways via regulating oxidative stress, etc. Notably, it is first reported that NG may regulate osteoclast differentiation and oxidative stress through the expression of the core targets so as to treat OF.

## 1. Introduction

Osteoporosis (OP) is a bone disease that often results in severe consequences such as fracture [1, 2]. Osteoporotic fracture (OF) is one of the most serious outcomes and clinical endpoints of OP because the lifetime risk of any OF is very high, ranging from 40 to 50 percent for women and 13 to 22 percent for men [3]. In accordance with a Chinese report in 2015, there were about 2.69 million cases of OF happening mainly in the wrists, hips, and vertebral body. OF seriously endangers the life and health of the elderly and increases the burden on families and society. OF is mainly treated using drugs for inhibition of bone resorption in clinics, but the clinical application of these drugs is limited due to some complications for long-term use [4]. Recently, traditional Chinese medicine has been gradually proved to have a functional effect on treating OF, which has attracted increasing attention from more and more scholars [5].

*Drynariae Rhizoma* (Chinese name: Gusuibu) is widely used to prevent and treat OF and OF-related bone diseases, whose isolated active constituents are composed of flavonoids, phenolic acids, triterpenes, and their glycosides [6]. Among them, flavonoids are the hot spots of current research on the active constituents of *Drynariae Rhizoma*. It has been reported that total flavonoids of *Drynariae Rhizoma* can reduce the production of reactive oxygen species (ROS) to alleviate osteoporosis [7]. Naringin (PubChem CID: 442428), the main ingredient of the flavonoids from *Drynariae Rhizoma*, has the curative effect of treating osteoporosis and promoting fracture healing, so it has a good application prospect in clinics [6]. Current studies have revealed that NG can promote osteoblast proliferation [8]. Moreover, NG can promote osteogenic differentiation and fracture healing by inducing the expression of bone morphogenetic protein-2 (BMP-2) [9]. However, multiple targets and pathways are involved in the process of NG treating OF, and it is hard for traditional pharmacology to carry out systematic analysis about its complex underlying mechanism.

Oxidative stress (OS) is considered to be one of the most critical pathogenic factors of age-related bone loss, which is a primary factor in OF; oxidative stress and bone loss increase with aging, thus leading to OF [10]. Studies have reported that NG has a protective effect against bone loss through relieving oxidative stress [11, 12]. To our knowledge, data on NG treating OF through relieving oxidative stress are scanty, so the correlation between oxidative stress and NG in treating OF is worth further exploring.

For better understanding the potential mechanism of NG treatment on OF, we adopted an integrative strategy of network pharmacology and molecular docking [13], which would provide a profound theoretical basis for NG application in treating OF.

## 2. Materials and Methods

### 2.1. Network Pharmacological Data Screening

#### 2.1.1. Naringin-Related Structure and Target Proteins

Naringin-related structure and targets were obtained in 3 steps. Step 1: Data retrieval was performed with the TCMSP database (https://tcmsp-e.com/) [14]. NG was retrieved from the “Chemical name” search box in the TCMSP database to obtain the structure and target information. The advantage of this approach is that the TCMSP database provides a comprehensive study for naringin. Step 2: The structure of naringin was exported as an “SDF” file by retrieving the PubChem database (https://pubchem.ncbi.nlm.nih.gov/), which was input into the SwissTargetPrediction database (http://new.swisstargetprediction.ch/) [15] to obtain the naringin-related targets. Step 3: The target proteins of naringin obtained from the two databases were imported into the UniProt database (http://www.uniprot.org/uniprot/), and the “popular organisms” search field was set to humans, so that target proteins of naringin were obtained, which were referred to as gene symbols.

#### 2.1.2. OF-Related Genes and Corresponding Proteins

Genes of OF were obtained from 2 databases (GeneCards and Online Mendelian Inheritance in Man). GeneCards, a human gene database (https://www.genecards.org/) [16], includes more than 190 data sources about genes, diseases, pathways, and compounds. Online Mendelian Inheritance in Man (OMIM, https://omim.org/) [17] contains information on all known Mendelian disorders and over 15,000 genes. The same key word “Osteoporotic Fracture” was searched in the two databases, and the species was limited to “*Homo sapiens*.” The proteins corresponding to OF-related genes were standardized using the UniProt database for subsequent analysis.

#### 2.1.3. Intersection Target Proteins (ITPs)

We used R (v3.6.1) software (Statistics Department of the University of Auckland, New Zealand) to take the intersection between OF-related proteins and the target proteins of NG to obtain ITPs.

### 2.2. Network Pharmacological Data Analysis

#### 2.2.1. Protein-Protein Interaction (PPI) Analysis

To explore the interaction between ITPs, we adopted the STRING database (https://string-db.org/) [18] to obtain the PPI data, which was exported as a .tsv file for further analysis. Next, the PPI information of intersection target proteins was used for the following work by using Cytoscape (v3.7.2) software (Institute for Systems Biology, Seattle, Washington, USA; http://www.cytoscape.org/) [19]. Briefly, the NG-intersection target-OF network was generated. Afterward, we constructed the PPI network of ITPs and performed a network topology analysis by using the Cytoscape plugin NetworkAnalyzer to count the degrees. The target proteins, whose degrees were above average, were considered core target proteins.

#### 2.2.2. GO Enrichment Analysis and KEGG Pathway Analysis

GO and KEGG analyses of the intersection targets were performed using clusterProfiler package (R3.6.1). The enrichment results with *P* < 0.05 were extracted.

### 2.3. Molecular Docking between Core Targets and Naringin

AutoDock Vina (v1.1.2) software (Department of Molecular Biology, The Scripps Research Institute, La Jolla, California, USA) [20] was utilized to conduct molecular docking simulations on naringin and its core targets to verify its interaction activity. The 3D structure of naringin was downloaded from the PubChem database (https://pubchem.ncbi.nlm.nih.gov/). AutoDockTools (v1.5.6; Department of Molecular Biology, The Scripps Research Institute, La Jolla, California, USA) was used to combine nonpolar hydrogen and distribute charge for naringin, the results of which were converted into a PDBQT file. The crystal structures of proteins were obtained from the RCSB PDB website (http://www.rcsb.org/), and the selection of proteins should satisfy the following three principles: (i) it should be a human protein; (ii) the protein owns one or more eutectic ligands, and the eutectic ligand having higher structural similarity with naringin is preferred; and (iii) the crystal structure with smaller “resolution” value is selected. Molecular docking simulations were not conducted, when the eutectic ligand structure of the protein could not be found in the PDB database or its specific active sites could not be obtained through literature search. We used AutoDockTools to separate the target protein from its ligand, add polar hydrogen and distribute charge, and then output the results to a PDBQT file. AutoDockTools was also used to determine the size and center of the docking box. The specific process of docking is as follows: First, the eutectic ligand of target protein was used to obtain the affinity of the docked protein as the comparison of the docking results of naringin. Then, naringin was successively docked with the target proteins and every affinity was calculated. Finally, the docking results of naringin were plotted and analyzed using PyMOL software (DeLano Scientific Limited Liability Company, South San Francisco, USA).

## 3. Results

### 3.1. The Structure and Target Proteins of Naringin

Finally, 105 targets of naringin were obtained from TCMSP and SwissTargetPrediction databases. After input into the UniProt database, target proteins of naringin were obtained, which were referred to as gene symbols. The structure and target information of naringin are shown in Supplementary Tables S1 and S2.

### 3.2. OF-Related Target Proteins and ITPs

**Figure 1:**
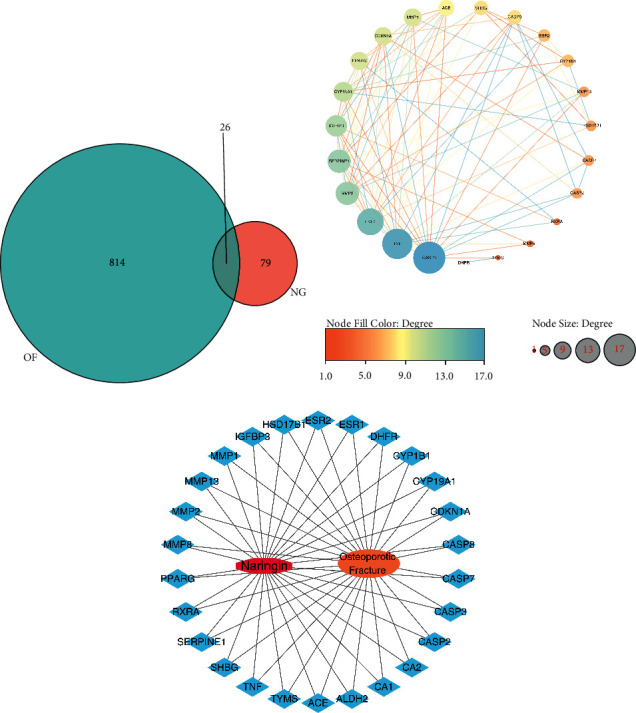
Venn diagram of NG-OF intersection targets: (a), PPI network of potential targets (b), and NG-intersection target-OF network (c).

Totally 840 target proteins of OF were retrieved in GeneCards and OMIM databases. After they were mapped with the target proteins of naringin, ITPS were obtained including 26 intersection targets, which are shown in Figure 1(a) and Table 1.

### 3.3. PPI Network Plotting and Core Target Protein Identification

We input ITPs into the STRING database, hiding the targets with no interactive relationship with others. And then, the data of protein-protein interaction (PPI) were obtained, which was imported into Cytoscape (v 3.7.2) to plot the PPI network in Figure 1(b). There were 11 target proteins whose degrees were higher than the average degree (7.91), which were predicted as the core target proteins (Table 2).

### 3.4. NG-Intersection Target-OF Network Construction

Figure 1(c) shows the NG-intersection target-OF network, which involved 28 nodes and 52 edges, including one NG node, one OF node, and twenty-six target nodes. In Figure 1(c), the blue diamond nodes represent the intersection target proteins. The red polygon node represents “naringin.” The orange oval node represents “osteoporotic fracture.” The edges represent the corresponding relationship among naringin, osteoporotic fracture, and the intersection targets.

### 3.5. GO Enrichment Analysis

**Figure 2:**
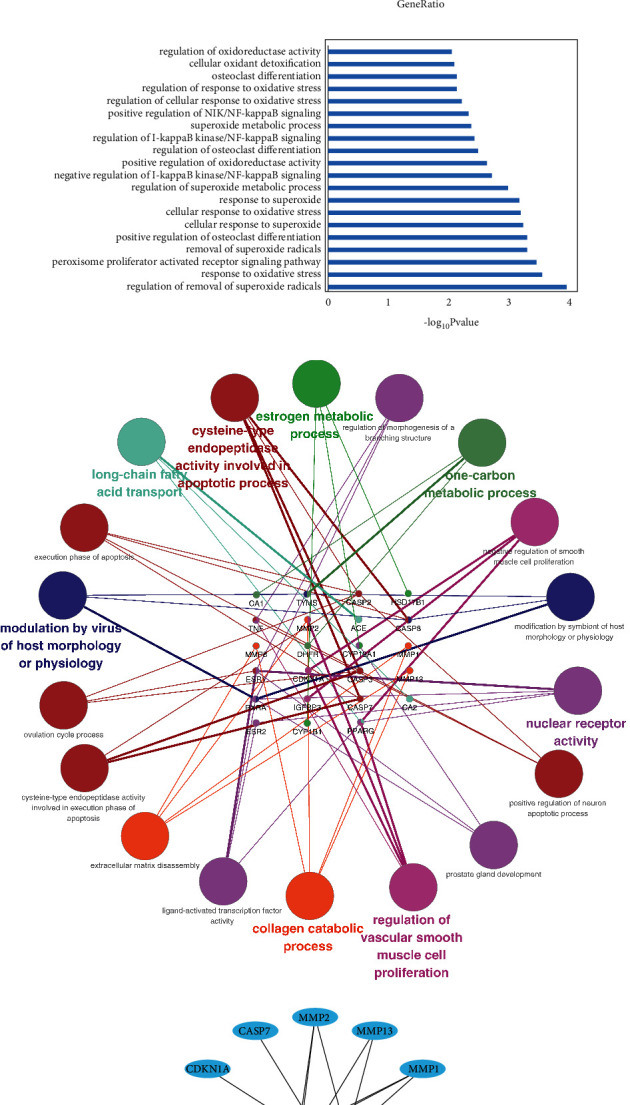
GO.BP enrichment analysis (a, b, c) and pathway-target network (d).

We obtained 415 items of biological process (BP).  Figure 2(a) shows the top 20 items. Notably, we have screened 20 items mainly related to oxidative stress, osteoclast differentiation, and NF-кB signaling regulations, which are shown in Figure 2(b). In addition, 26 interaction targets were imported into Cytoscape (v3.7.2) for GO.BP enrichment analysis. The GO.BP results are mainly related to the following aspects shown in Figure 2(c): (i) inflammation-related activities, such as nuclear receptor activity and extracellular matrix disassembly, which are closely associated with oxidative stress; (ii) cell cycle, such as execution phase of apoptosis; (iii) hormone metabolism, such as estrogen metabolism process; and (iv) lipid metabolism, such as long-chain fatty acid transportation.

### 3.6. KEGG Pathway Analysis

The KEGG pathway analysis of 26 target genes was performed using R software. A total of 37 items were obtained, and Table 3 lists 7 key signaling pathways. Cytoscape (v3.7.2) was used for network visualization, as shown in Figure 2(d).

### 3.7. Molecular Docking Analysis

Among 11 core targets, a total of 8 proteins suitable for molecular docking were obtained after screening, including ESR1, CASP3, ACE, TNF, PPARG, SERPINE1, CYP19A1, and MMP1. To verify how naringin binds to core targets as previously referred, molecular docking using AutoDock Vina was developed in this section. We predicted whether naringin could enter the active pocket of target proteins successfully or not and calculated the affinities between them. The relevant information of the eight proteins and the docking results of eutectic ligands are shown in Supplementary Table S3, and the affinity and hydrogen bond information of naringin with each target protein are shown in Table 4.

**Figure 3:**
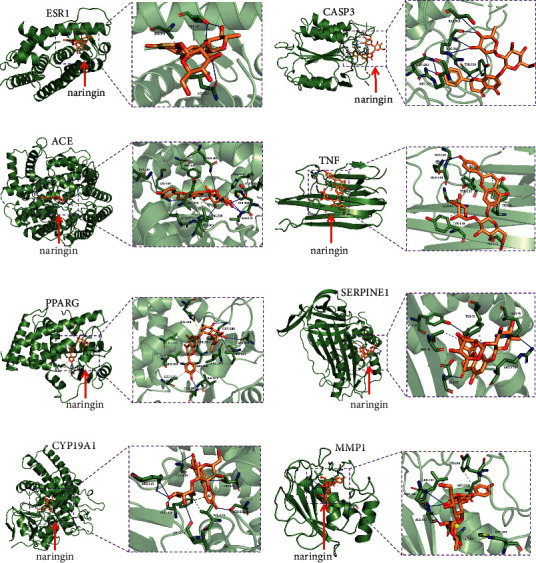
Simulated molecular docking of naringin on ESR1: (a), CASP3 (b), ACE (c), TNF (d), PPARG (e), SERPINE1 (f), CYP19A1 (g), and MMP1 (h).

As presented in Table 4 and Figure 3(a), the binding affinity of this combination is −7.1 kcal/mol. Naringin was bound with ESR1 by forming 2 hydrogen bonds with Thr-347, while one hydrogen bond with Leu-525. In addition, there are hydrophobic contacts between naringin and Ala-350 and Leu-525.

As presented in Table 4 and Figure 3(b), the binding affinity of naringin upon CASP3 is −5.4 kcal/mol. The residues including Arg-179, Gln-283, Tyr-338, Arg-341, and Ser-343 interact with naringin by forming 9 hydrogen bonds, which provide a powerful electrostatic force for the combination of naringin and CASP3.

As presented in Table 4 and Figure 3(c), the binding affinity of naringin upon ACE was −9.4 kcal/mol. There were 8 hydrogen bonds provided by Glu-162, Gln-281, His-383, His-513, Lys-449, Lys-454, and Ala-354 residues in the interaction with naringin. Naringin was located in the hydrophobic pocket comprising Glu-376, Val-379, Val-380, and Phe-457 residues. Interestingly, naringin interacts with the His-353 residue by salt bridge.

As presented in Table 4 and Figure 3(d), the binding affinity of naringin upon TNF was −6.5 kcal/mol. There were hydrogen bonds provided by Gly-121, Gly-148, and Gln-149 residues in the interaction with naringin. Naringin was located in the hydrophobic pocket comprising Tyr-59, Gln-61, and Tyr-119 residues. Interestingly, naringin interacts with Tyr-59 and Tyr-151 residues by Pi-Pi stacked interactions.

As presented in Table 4 and Figure 3(e), the binding affinity of naringin upon PPARG was −9.1 kcal/mol. There were 6 hydrogen bonds provided by Ala-278, Ile-281, Gln-286, Ser-289, and Glu-343 residues in the interaction with naringin. Naringin was located in the hydrophobic pocket of PPARG. Interestingly, naringin interacts with the His-449 residue by salt bridge.

As presented in Table 4 and Figure 3(f), the binding affinity of naringin upon SERPINE1 was −7.2 kcal/mol. There were hydrogen bonds provided by Tyr-37, Arg-76, Tyr-79, Asp-95, and Arg-118 residues in the interaction with naringin. Tyr-79, Thr-93, and Arg-118 residues interact with naringin by hydrophobic interaction.

As presented in Table 4 and Figure 3(g), the binding affinity of naringin on CYP19A1 was −9.0 kcal/mol. There were 7 hydrogen bonds provided by Thr-310, Ser-314, Leu-372, Phe-430, Gly-439, and Arg-115 residues in the interaction with naringin. In addition, naringin interacts with the Phe-430 residue by T-type Pi-Pi stacked interaction.

As presented in Table 4 and Figure 3(h), the binding affinity of naringin upon MMP1 was −9.5 kcal/mol. The residues including Asn-180, Leu-181, Glu-219, and Ala-182 interact with naringin by forming 5 hydrogen bonds, which provide a powerful electrostatic force for the combination of naringin and MMP1. Interestingly, naringin interacts with the His-228 residue by salt bridge.

## 4. Discussion

Chinese traditional medicine *Drynariae Rhizoma* has been widely applied in treating osteoporosis and osteoporotic fracture clinically for many years. Naringin is one of the most abundant flavonoids in *Drynariae Rhizoma*, which is also found in citrus fruits and many other Chinese medicines [6]. Studies have revealed that flavonoids have the therapeutic effect on OF by regulating estrogen receptor, OPG/RANK/RANKL, enzyme inhibition, or signal transduction pathways [21, 22].

In this study, there were 26 common targets obtained between NG and OF, among which 11 core targets were identified, namely CASP3, TNF, ESR1, MMP2, MMP1, SERPINE1, IGFBP3, CYP19A1, PPARG, CDKN1A, and ACE. PPI network topology analysis suggested that the targets were mainly characterized by oxidative stress, inflammation, cell cycle, and lipid and hormone metabolism-related proteins. The top three targets in terms of degree are TNF, CASP3, and ESR1, indicating that they may be the key targets of NG in the treatment of OF.

TNF (tumor necrosis factor), known as TNF-*α*, which is the earliest inflammatory medium produced in oxidative stress response, can promote the generation of inflammatory mediators and induce macrophage colony-stimulating factor (M-CSF) expression [23]. TNF can increase bone absorption and thus influence the healing of OF through activating NF-кB and promoting RANKL-induced osteoclast differentiation [24]. TNF-*α* can promote oxidative stress and regulate bone homeostasis and bone reconstruction [25, 26]. Moreover, it has been reported that NG could decrease the TNF-*α* expression [27]. Therefore, we speculated that NG could reduce oxidative stress by downregulating TNF expression in OF patients, so as to anti-OF.

CASP3 (caspase-3) affects the apoptosis of osteoclasts [28]. Some studies have proved that the upregulation of CASP3 mRNA can promote OF healing [29]. Further studies have shown that the upregulation of CASP3 can activate the p53 signaling pathway, destroy the maturation of osteoblasts, and inhibit chondrocyte differentiation, thus promoting fracture healing [30].

ESR1 (estrogen receptor 1) has a close relationship with bone formation [31]. When ESR1 is combined with estrogen, it can up- or downregulate cytokines to affect relevant signaling pathways. ESR1 affects the proliferation, differentiation, and maturation of chondrocytes, regulates the process of endochondral osteogenesis, and maintains chondrocyte phenotype, which promotes the maintenance of cartilage thickness, bone growth balance, and fracture healing [32]. The lack of estrogen results in the acceleration of bone mass loss in postmenopausal women, which could easily give rise to OF [33]. Estrogen exerts physiological activities in cells through ESR1, which are mainly manifested in cell growth, differentiation, senescence, and apoptosis [34]. Guo et al. [35] reported that naringin from *Drynariae Rhizoma* revealed a double directional adjusting function of estrogenic and antiestrogenic activities. Pang et al. [6] also demonstrated that naringin might mediate ligand-independent activation of ESR1 in osteoblastic cells to protect against ovariectomy-induced bone loss in mice. However, studies are needed to determine whether naringin could prevent and treat OF via exerting estrogen-like protective actions in bone.

MMP2 (matrix metalloproteinase 2) and MMP1 (matrix metalloproteinase 1) are all members of the family of matrix metalloproteinases (MMPs). MMP2 is expressed in the cytoplasm of osteoblasts and some osteoclasts, which can regulate the dissolution of bone matrix, inhibit bone resorption, and contribute to bone reconstruction [36], which promotes fracture healing. MMP1 is closely related to the repair of cartilage tissue. It has been shown that the expression of MMP1 can inhibit the degradation of cartilage matrix and promote the repair of cartilage [37]. Nevertheless, there is no research revealing the regulatory function of NG on MMP2 or MMP1 in OF, which is needed to be further studied in the next step study.

PPARG (peroxisome proliferator-activated receptor gamma), which is related to the regulation of cell differentiation, inflammatory response, and oxidative stress, mainly affects the catabolism of lipids and plays a key role in the process of adipocyte differentiation [38]. It can promote lipid formation and inhibit osteogenesis through different regulatory pathways like PPAR*γ*2 signaling pathway [39, 40]. A study [41] reported that naringin can protect against steroid-induced avascular necrosis of the femoral head (SANFH) through upregulation of PPAR*γ*2 and activation of the Notch signaling pathway in a rabbit model.

CDKN1A (cyclin-dependent kinase inhibitor 1), also known as p21, is a negative regulator of cell cycle that regulates cell proliferation, differentiation, and senescence [42]. The number of osteoblasts is closely related to cell proliferation, and the process of cell proliferation cycle is mainly regulated by cycle-regulating proteins, of which the regulation of *G*1 phase is the most important [43]. In this process, the combination of cyclin *D*1 and CDK4 (cyclin-dependent kinase 4) forms the CDK4-cyclin complex to promote the *G*1 phase [44]. CDKN1A (p21), as a *G*1 phase regulatory protein, inactivates the CDK4-cyclin complex by binding to it, leading to cell cycle stagnation in the *G*1 phase [45]. An animal experiment [46] has proved that the expression of p21 in osteoblasts was significantly increased in rats after ovariectomy (*P* < 0.01). It is also reported that the downregulation of p21 protein expression can promote the proliferation of osteoblasts [45]. Thus, p21 plays a key role in the proliferation of osteoblasts and NG might treat OF by upregulating the p21 expression, which is expected to be demonstrated in the future research.

In addition, CASP2, CASP3, CASP7, and CASP8 in Table 3 are members of the cysteine protease family, which can promote osteoclast apoptosis and thus promote fracture healing [47, 48]. ESR2 is also a receptor of estrogen, which is associated with postmenopausal OF [33].

The results of GO enrichment analysis are similar to those of PPI network analysis. Notably, GO.BP enrichment analysis reveals that the regulation of oxidative stress and osteoclast differentiation play a critical role in NG treating OF, as shown in Figure 2(b). In recent years, relevant reports have confirmed that oxidative stress plays a key role in the pathogenesis of OF [49]. Studies have also revealed that oxidative stress (OS) is one of the important factors that trigger OF [50, 51]. OS is due to the generation of excess reactive oxygen species (ROS), which cannot be cleared by endogenous antioxidants, exceeding the normal physiological threshold and triggering a series of cell toxic reactions, thus further causing tissue damage [52]. Oxidative stress induced by ROS can cause changes in bone homeostasis, increase bone resorption, and decrease bone formation, leading to the occurrence of OF [53]. Some scholars have reported that miR-320a can increase the OS level, reduce osteoblast function, and result in the occurrence of OF [54]. Liu et al. [55] found that the reduction of ROS can increase bone mass and prevent ovariectomized osteoporosis. And some studies have revealed the protective effects of naringin against H2O2-induced inhibition of osteogenic differentiation, which suggests that naringin is a natural antioxidant [56]. Based on our study, naringin may be a promising antioxidant, which would help relieve oxidative stress and ameliorate OF. Moreover, numerous studies have demonstrated that the expression of core targets including TNF [57], CASP3 [58], ESR1 [59], MMP2 [60], MMP1 [61], PPARG [62], CDKN1A [63], ACE [64], etc., plays an important part in regulating oxidative stress. Therefore, we speculated that NG could regulate core targets' expressions and osteoclast differentiation by oxidative stress in OF patients, so as to anti-OF.

KEGG pathway enrichment analysis demonstrated that p53, IL-17, TNF, estrogen, and PPAR signaling pathways may play a key role in naringin treating OF. Similarly, the results of KEGG analysis are consistent with those of PPI and GO analysis.

Some studies have verified that the activation of p53 signaling pathway can destroy the maturation of osteoblasts and inhibit chondrocyte differentiation [30]. Studies have shown that NG could inhibit inflammation and apoptosis mediated by p53, NF-кB, and TNF pathways [65, 66]. However, whether NG could regulate the p53 signaling pathway to treat OF is still unclear, which needs further identification in the future research.

The IL-17 signaling pathway can stimulate the synthesis of TNF-*α*, IL-6, and NF-кB, thus promoting osteoclast differentiation induced by RANKL [67]. Moreover, studies have shown that the TNF signaling pathway plays a critical role in the occurrence of postmenopausal OF through promoting the expression of RANKL and inducing osteoclast differentiation [23]. So, IL-17 [68] and TNF signaling pathways [69] have close connection with osteoclast differentiation.

The estrogen signaling pathway can regulate the proliferation, differentiation, and apoptosis of osteoblasts and osteoclasts [70]. It has also been proved that the PPAR pathway can inhibit the lipogenesis of bone marrow, promote the generation of osteoblasts, and improve bone formation and bone mass [40], which promotes fracture healing.

Collectively, the results in our study predicted some pathways and targets that may be potentially therapeutic targets and provide reference for future research on NG treating OF. Nevertheless, a limitation of this study is that further experiments are necessary to demonstrate our findings.

## 5. Conclusion

In summary, for the first time, our results revealed that naringin may treat OF possibly by regulating numerous signaling pathways and targets related to oxidative stress and osteoclast differentiation. These results will provide a theoretical basis for the treatment of OF. However, these predicted altered signaling pathways or target genes still need to be further verified in the future study.

## Figures and Tables

**Table 1:** Potential target genes of NG in the treatment of OF.

Number	Gene	Number	Gene
1	CDKN1A	14	ALDH2
2	TNF	15	CASP3
3	CYP19A1	16	ACE
4	CA2	17	PPARG
5	CA1	18	IGFBP3
6	MMP1	19	CASP7
7	MMP8	20	CASP8
8	MMP13	21	CASP2
9	CYP1B1	22	RXRA
10	HSD17B1	23	SERPINE1
11	SHBG	24	DHFR
12	ESR1	25	MMP2
13	ESR2	26	TYMS

**Table 2:** Core targets of NG in the treatment of OF.

Number	Core targets	Degree
1	CASP3	17
2	TNF	16
3	ESR1	14
4	MMP2	12
5	SERPINE1	12
6	IGFBP3	11
7	CYP19A1	10
8	PPARG	9
9	CDKN1A	9
10	MMP1	9
11	ACE	8

**Table 3:** KEGG pathway enrichment analysis.

ID	Signaling Pathway	Enriched Gene Number	*P* value
hsa04115	p53 Signaling Pathway	5	0.000271
hsa04657	IL-17 Signaling Pathway	5	0.000508
hsa04933	AGE-RAGE Signaling Pathway	4	0.002583
hsa04668	TNF Signaling Pathway	4	0.00296
hsa03320	PPAR Signaling Pathway	3	0.009397
hsa04926	Relaxin Signaling Pathway	3	0.028958
hsa04915	Estrogen Signaling Pathway	3	0.032836

**Table 4:** Molecular interactions of core targets and naringin.

Compound	Target	Affinity (kcal/mol)	Number of hydrogen bonds	Hydrogen bonds interacting residues
Naringin	ESR1	−7.1	3	Thr-347(2), Leu-525
Naringin	CASP3	−5.4	9	Arg-179, Gln-283, Tyr-338(2), Arg-341(4), Ser-343
Naringin	ACE	−9.4	8	Glu-162, Gln-281, Ala-354(2), His-383, Lys-449, Lys-454, His-513
Naringin	TNF	−6.5	3	Gly-121, Gly-148, Gln-149
Naringin	PPARG	−9.1	6	Ala-278, Ile-281, Gln-286, Ser-289(2), Glu-343
Naringin	SERPINE1	−7.2	5	Tyr-37, Arg-76, Tyr-79, Asp-95, Arg-118
Naringin	CYP19A1	−9.0	7	Arg-115(2), Thr-310, Ser-314, Leu-372, Phe-430, Gly-439
Naringin	MMP1	−9.5	5	Asn-180, Leu-181, Ala-182(2), Glu-219

## Data Availability

The data used to support the study's results came from the first author.

## Supplementary Materials

Supplementary Materials Supplementary Table S1: the structure of naringin. Supplementary Table S2: targets of naringin. Supplementary Table S3: target protein information and original ligand docking results.
