# Supplementary material for: Network Pharmacology Integrated with Molecular Docking Explores the Mechanisms of Naringin against Osteoporotic Fracture by Regulating Oxidative Stress
Source: Evid Based Complement Alternat Med. 2021 Sep 20;2021:6421122. doi: 10.1155/2021/6421122 (PMC8476256; doi:10.1155/2021/6421122)
Supplement: Supplementary Materials — Supplementary Table S1: the structure of naringin. Supplementary Table S2: targets of naringin. Supplementary Table S3: target protein information and original ligand docking results. [file 6421122.f1.docx]

**Supplementary Table S1. The Structure of Naringin**

| Molecule ID | PubChem CID | Molecule name | Chemical formula | Structure | OB（%） | DL |
| --- | --- | --- | --- | --- | --- | --- |
| MOL005812 | 442428 | naringin | C_27_H_32_O_14_ | 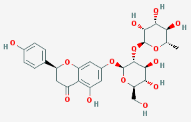 | 6.92 | 0.78 |

OB: oral bioavailability; DL: drug-likeness

**Supplementary Table S2. Targets of Naringin**

| Number | Gene symbol | Number | Gene symbol | Number | Gene symbol |
| --- | --- | --- | --- | --- | --- |
| 1 | TOP2A | 36 | EIF4A1 | 71 | GRM2 |
| 2 | CDKN1A | 37 | PTPN2 | 72 | LGALS7 |
| 3 | TNF | 38 | CA3 | 73 | FOLH1 |
| 4 | RASGRF2 | 39 | CA6 | 74 | CES1 |
| 5 | RAF1 | 40 | CA13 | 75 | PPARG |
| 6 | CYP19A1 | 41 | CA5B | 76 | CES2 |
| 7 | SLC5A1 | 42 | ABCB1 | 77 | MME |
| 8 | SLC5A4 | 43 | CA5A | 78 | ECE1 |
| 9 | SLC5A2 | 44 | CA7 | 79 | PLA2G1B |
| 10 | SRD5A1 | 45 | CA4 | 80 | IGFBP3 |
| 11 | EPHX2 | 46 | ESR1 | 81 | CASP6 |
| 12 | TYR | 47 | ESR2 | 82 | CASP7 |
| 13 | SLC28A3 | 48 | LGALS1 | 83 | CASP8 |
| 14 | CA12 | 49 | VARS | 84 | CASP1 |
| 15 | CA2 | 50 | LARS | 85 | CASP2 |
| 16 | CA1 | 51 | CHIA | 86 | RXRA |
| 17 | MMP1 | 52 | PARP1 | 87 | IRAK4 |
| 18 | MMP7 | 53 | PTPN1 | 88 | HRAS |
| 19 | MMP8 | 54 | ADORA2B | 89 | CDK1 |
| 20 | ADORA1 | 55 | TDP1 | 90 | NEU4 |
| 21 | ADORA3 | 56 | ALDH2 | 91 | SLC28A2 |
| 22 | TAS2R31 | 57 | FHIT | 92 | HCAR2 |
| 23 | CA14 | 58 | CASP3 | 93 | SERPINE1 |
| 24 | MMP13 | 59 | AKR1B1 | 94 | DHFR |
| 25 | MMP12 | 60 | ACE | 95 | MMP2 |
| 26 | IMPDH1 | 61 | SLC29A1 | 96 | CHEK1 |
| 27 | MAOB | 62 | GAA | 97 | GRM5 |
| 28 | CYP1B1 | 63 | ABL1 | 98 | TACR2 |
| 29 | ABCG2 | 64 | AMY2A | 99 | POLB |
| 30 | PTGS1 | 65 | EPHA2 | 100 | PDE5A |
| 31 | ADORA2A | 66 | PYGL | 101 | KLK1 |
| 32 | ABCC1 | 67 | LCK | 102 | KLK2 |
| 33 | HSD17B1 | 68 | MAP3K9 | 103 | SLC10A2 |
| 34 | SHBG | 69 | BTK | 104 | LGALS4 |
| 35 | CBR1 | 70 | EDNRA | 105 | TYMS |

**Supplementary Table S3. Target Protein Information and Original Ligand Docking Results**

| Gene | Protein | PDB ID | Ligand ID | Affinity(kcal/mol) |
| --- | --- | --- | --- | --- |
| ESR1 | Estrogen receptor | 1ERR | RAL | -10.3 |
| CASP3 | Caspase-3 | 1RE1 | NA3 | -4.8 |
| ACE | Angiotensin-converting enzyme | 1UZF | MCO | -5.8 |
| TNF | Tumor necrosis factor | 2AZ5 | 307 | -6.8 |
| PPARG | Peroxisome proliferator-activated receptor gamma | 2G0H | SP3 | -11.7 |
| SERPINE1 | Plasminogen activator inhibitor 1 | 4AQH | TB7 | -7.7 |
| CYP19A1 | Cytochrome P450 19A1 | 4GL5 | G29 | -10.5 |
| MMP1 | Matrix metalloproteinase 1 | 966C | RS2 | -9.5 |
